# Intraosseous Catheter Flow Rates and Ease of Placement at Various Sites in Canine Cadavers

**DOI:** 10.3389/fvets.2019.00312

**Published:** 2019-09-19

**Authors:** James Lange, Søren R. Boysen, Adam Bentley, Aylin Atilla

**Affiliations:** ^1^Department of Veterinary Clinical and Diagnostic Sciences, Faculty of Veterinary Medicine, University of Calgary, Calgary, AB, Canada; ^2^VCA Canada Western Veterinary Specialist and Emergency Centre, Calgary, AB, Canada

**Keywords:** intraosseous, flow rates, fluid therapy, canine, veterinary

## Abstract

**Objective:** To compare intraosseous catheter placement difficulty, success rates, and flow rates at four different locations in canine cadavers.

**Design:** Prospective study.

**Setting:** Private referral center.

**Animals:** Eleven fresh canine cadavers.

**Interventions:** With owner consent, animals presenting for euthanasia were recruited. Animals received heparin (1,000 IU/kg IV) at least 5 min prior to euthanasia. After euthanasia, EZIO intraosseous catheters were placed into the ilial wing, proximal medial tibia, proximal lateral humerus, and distal lateral femur on one side of the animal. Time to catheter placement and catheter difficulty were scored for each placement site. Sterile saline was infused into each location simultaneously over 5 min, first via gravity then using 300 mmHg pressure. Animals were repositioned onto the contra-lateral side and the experiment repeated.

**Measurements and Main Results:** Placement was successful in 16/22 ilial, 18/22 tibial, and 22/22 femoral and humoral attempts. A *post-hoc* analysis revealed the ileum had a significantly greater difficulty score when compared to the femur and humerus (*p* ≤ 0.0001). The femur had a statistically significant faster placement time when compared to the ileum (*p* ≤ 0.05). Gravity infusion rates were statistically lower in the tibia when compared to humerus (*p* ≤ 0.01) and between the tibia when compared to the femur (*p* ≤0.001). Additionally, pressurized infusion rates were statistically lower in the tibia compared to the humerus (*p* ≤ 0.0001), the femur (*p* ≤ 0.0001), and the ileum (*p* ≤ 0.01).

**Conclusions:** The femur and humerus had high success rate for IO catheter placement and low placement time and difficulty scores. Pressurized intraosseous flow rates were highest in the humerus and femur. Contrary to human literature, success rates for catheter placement in the humerus and femur were higher than at other sites, suggesting the humerus and femur may be preferred sites for intraosseous catheter placement in the dog. Further investigation through a larger sample size is required to confirm these findings.

## Introduction

Intraosseous (IO) catheterization was first described as two independent discoveries by Drinker et al. and Doan in 1922, as a means of gaining vascular access in human patients (1, 2). While the use of IO catheters diminished with the invention of plastic intravenous catheters (3), there was a resurgence in IO catheter utilization in the 1980's, as it was recognized as an effective means of gaining vascular access in hypovolemic pediatric human patients (4). Since then, there has been growing recognition that IO catheters provide a rapid and consistent means of vascular access in patients of all ages. Factors such as trauma, peripheral edema, small patient size, and obesity can also increase the difficulty of intravenous (IV) access (5). A recent meta-analysis comparing adults that received epinephrine via IV or IO catheters in out of hospital cardiac arrest scenarios demonstrated that IO catheters provide equivalent return of spontaneous circulation when compared to percutaneous IV catheters (6). Interestingly, there was a higher successful first attempt placement in the IO group when compared to the IV group.

Achieving rapid vascular access through the peripheral venous system can be complicated by factors such as hypovolemia, cardiovascular collapse, cardiovascular arrest, and traumatic injuries such as fractures, abrasions, and degloving injuries. Often peripheral IV access in critically ill patients can be difficult to achieve due to the hypotensive effects of shock or peripheral vasoconstriction (7). An alternative route of IV access is to perform a venous cut-down, often over the jugular vein, as this vessel allows rapid infusion of fluids for resuscitation (8). However, studies in human medicine have shown that IO catheterization takes less time, has lower failure rates and less complications than central venous catheters (8, 9). A similar study in canine cadavers demonstrated that placement time for IO catheter was significantly faster when compared to jugular venous catheterization (10). Both human and veterinary literature has consistently demonstrated that IO access offers a rapid, non-collapsible, and safe route for fluid and drug administration in patients where standard peripheral catheterization is difficult or impossible.

The renewed interest in IO catheter utility has led to many studies comparing the different anatomical locations for IO catheter placement using human and animal models (11–15). The trochanteric fossa of the femur is often used in veterinary exotic pets and pediatric patients, however varying amounts of soft tissue covering the trochanteric fossa as well as nearby structures such as the sciatic nerve may dissuade operators from placing an IO catheter at this location. The tibia has previously been reported as the preferred site for IO catheter placement for the resuscitation of adult cats and dogs (16). A recent adult cadaver study demonstrated that the proximal humerus and sternum facilitate the highest fluid flow rates when compared to the tibia and thus may be the preferred anatomical sites for rapid resuscitation and drug administration in people (13). This new insight goes against the standard clinical practice as the tibia is often the chosen site due to the ease of landmark location and lack of overlying muscle/tissue to traverse when passing an IO catheter (5, 13). At present, there are no studies in the veterinary literature that investigate infusion rates of IO catheters in different anatomical sites in companion animals. Furthermore, data comparing the ease of placement between various anatomical sites in veterinary patients is restricted to juvenile canine, porcine, and bovine subjects (11, 12, 17).

The purpose of this canine cadaver study was to compare infusion rates, difficulty of placement, and success rates for IO catheterization in four clinically relevant locations: the proximal humerus, the distal lateral femur, the ileal wing, and the proximal medial tibia. We hypothesize that canine IO catheterization flow rates would mimic those of human medicine, and that the humerus would have the highest infusion rates and highest first attempt success rate.

## Materials and Methods

This study was reviewed and received ethics clearance from the University of Calgary's Veterinary Science Animal Care Committee (File # AC-16-0216). Patients presented for humane euthanasia were recruited into this study. Owner consent was obtained for all patients prior to euthanasia.

### Patient Recruitment

A total of 11 dogs were recruited into the study. Ten client owned adult dogs that presented to a private veterinary referral center for euthanasia and one dog animal that presented to Municipal Animal Shelter were recruited into this study. Dogs <10 kg were excluded. Animals were euthanized for various medical concerns (Table 1). Exclusion criteria included animals with gross pathology that was felt to affect the bone selected IO insertion sites (fractures, osteomyelitis, neoplasia, etc.).

**Table 1:** Subjects recruited into study.

**Breed**	**Age (years)**	**Gender**	**Weight (kg)**	**Reason for euthanasia**
Dachshund	13	Male neutered	10.1	Intervertebral disk disease
Rottweiler	9	Female spayed	30.0	Seizure
Newfoundland	12	Male neutered	49.0	Laryngeal paralysis
Staffordshire Bull Terrier	8	Male neutered	30.8	Dermatological disease
Boxer cross	12	Female spayed	32.0	Hemoabdomen
Labrador cross	15	Male neutered	27.3	Seizure/vomiting
Border collie	3	Female spayed	11.4	Liver disease
Rottweiler cross	12	Female spayed	37.0	Collapse
Doberman	5	Female spayed	36.2	Congestive heart failure
Bichon Frise	15	Male neutered	10.1	Uncontrolled diabetes mellitus
Shepherd cross	2	Male neutered	22.0	Behavioral disorder

### Euthanasia Procedure

All animals received heparin (10,000 IU/mL) dosed at 1,000 IU/kg at least 5 min prior to euthanasia. Heparin was administered to prevent post mortem blood coagulation, which may affect fluid flow rates. Euthanasia was performed by the on-duty Emergency Room veterinarian using a commercially available euthanasia solution (Euthanol). Client visitation time prior to and post-euthanasia was not restricted.

### IO Catheter Placement Procedure

IO catheters were placed by emergency room veterinarians with between 1 and 3 years of clinical experience. Prior to this study, neither clinician had experience using IO catheters, however, both investigators were trained on EZIO placement using cadavers and were required to successfully demonstrate placement in all locations, under supervision of an ECC specialist with extensive EZIO catheter placement experience, prior to starting the study.

**Figure 1:**
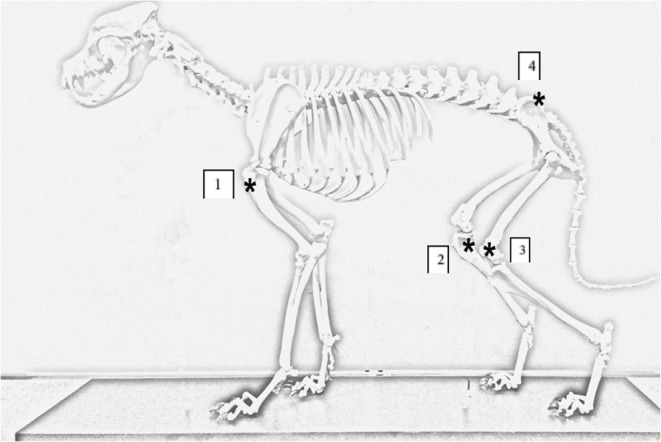
Canine skeleton demonstrating anatomical locations for IO catheter placement. Asterick annotates the IO catheter insertion sites on the canine cadaver. (1) Placement site for proximal humerus. (2) Placement site for proximal medial tibia. (3) Placement site for distal lateral femur. (4) Placement site for ileal wing.

Animals were placed in lateral recumbency, alternating the first side between patients (patient one was first placed in left lateral, patient two right lateral etc.). The order of IO placement rotated between each animal. Landmarks for each site were palpated and stab incisions with a number 15 scalpel blade were made over the site of IO catheter placement. Landmarks for each location were as follows (see Figure 1):

1. Proximal lateral humerus: greater tubercle, cranial aspect of the humerus, humeroscapular joint space, and the acromion process of the scapula; catheter was placed in the proximal epiphysis of the humerus.
2. Proximal medial tibia: tibial crest, caudal margin of the tibia, and stifle joint space; catheter was placed in the proximal epiphysis of the tibia.
3. Distal lateral femur: patella, fabella, and condyles of the femur; catheter was placed midway between the fabella and patella.
4. Wing of the ilium: the ileal wing was palpated and the catheter placed in the dorsocranial aspect directed caudoventral at an ~45° angle.

EZ-IO catheters (15 gauge, 304 stainless steel, 15 mm length (Mila International Inc.) were placed in each location using an EZ-IO Power Driver G3 (Mila International Inc., Kentucky, USA). IO catheters were placed in all four anatomical locations before infusions were started. Catheters were attached to a luer-lock syringe (Terumo Medical Corporation; Tokyo, Japan) containing 3 mL/kg of 0.9% NaCl, marrow and spicules were aspirated to confirm placement. Catheters were flushed with the 3 mL/kg 0.9% NaCl and attached to a 1 l bag of 0.9% NaCl (Fresenius Kabi, Toronto, Ontario) via a 15 drop/mL IV drip set, clamped closed using a single line clamp provided with the drip set. The top of the fluid bags were hung 100 cm from the base of each corresponding catheter. Placement was performed using the same IO catheters in the same manner as described above on the contralateral side.

### Time to Placement

Stab incisions were made prior to timing for placement and all materials required for IO catheter placement were situated next to the patient. The EZ-IO Power Driver G3 was pre-loaded with an EZ-IO catheter. The timer was started when the operator indicated they were ready to attempt placement. The timer was stopped when spicules and/or marrow were aspirated into the syringe (described above).

### Placement Difficulty

A scale was developed to assign a numerical value to the investigator's assessment of intraosseous catheter placement difficulty (Table 2). If initial placement of the intraosseous catheter failed to obtain marrow following initial placement, adjustments were made by hand, replacing the stylet and turning the catheter clock-wise to slowly back it out. If the catheter required further advancement the EZ-IO Power Driver G3 was reattached. A maximum of two syringe aspirations were allowed after initial catheter placement before readjustment was required. Two aspirations were also permitted after each readjustment attempt.

**Table 2:** Intraosseous catheter placement difficulty scores.

**Difficulty score**	**Score description**
1	Single placement attempt, no adjustment, immediate aspiration of marrow and/or spicules.
2	Aspiration of marrow and/or spicules after initial placement and one re-adjustment.
3	Aspiration of marrow and/or spicules after initial placement and two re-adjustments.
4	Aspiration of marrow and/or spicules after initial placement and three re-adjustments.
5	Complete failure. Failure to aspirate marrow and/or spicules after initial placement and three re-adjustments. OR accidental removal of catheter from bone during readjustments.

### Infusion Rates

Sodium chloride 0.9% was selected as the crystalloid for infusion. Bags were weighed on a gram scale before and after each period of infusion, the difference in weight was assumed to be equal to volume (1 g = 1 mL). First, infusion proceeded solely under gravity for exactly 5 min. The infusion was then stopped. Fluid bags were placed in pressure bags and re-hung 100 cm above the corresponding catheter site. The pressure bags were inflated to 300 mmHg. Infusion was again allowed to proceed for exactly 5 min. Line clamps were all simultaneously opened and closed at the start and end of each infusion period, respectively.

The weight of the fluid bags after infusion was subtracted from the weight prior to infusion to obtain a total volume infused. Infusion rates were calculated and standardized by dividing the total volume infused by the patient's body weight and the 5-min infusion period to give a value of mL/kg/min. Contralateral infusion rates were also performed and recorded in the same manner as described above.

## Data Analysis

### Infusion Rates

Scatter plots and a ROUT method (Q = 1%) were used to identify outliers. A Wilcoxon Matched Pairs Signed Rank Test was used to compare left and right sided data. A D'Agostino Pearson Omnibus test for normality was performed for gravity and pressure infusion at all anatomical sites. Normality was achieved at all four anatomical locations for both gravity and pressure infusions. A *p*-value of ≤ 0.05 was considered significant. Results are reported as mean ± standard deviation for data that passed normalcy and median and range for data that was not normally distributed. A *post-hoc* analysis one-way ANOVA Tukey Test was performed on data that passed normalacy and A Kruskal–Wallis One-way analysis of variance for non-parametric data and a *post-hoc* Dunn's Test was performed on data that failed to pass normalcy.

## Results

### Placement

Successful placement was achieved in 16 ileum, 18 tibia, 22 femurs, and 22 humerus. Dog 1 had failed placement on both tibia and both ileums. Dog 7 failed both tibia and one side of the ileum. Both of these animals were excluded from statistical analysis of flow rates.

Dog 10 had significant extravascularization of fluid in both tibia during pressurized infusion. As a result, both tibias were excluded from statistical analysis.

Dogs 2, 3, and 5 had one side (either left or right) excluded from analysis due to placement failure.

Dog 2 and Dog 3 each had one failed ileum placement. Dog 5 had one humerus and one ileum placement excluded from the study.

A total for 14 data points from each anatomical site was included in the final analysis for gravity and pressurized flow rates. A Wilcoxon Matched Pairs Signed Rank Test revealed no statistical difference between left and right side. Normality was not achieved with D'Agostino Pearson Omnibus test.

An ANOVA analysis requires all data points from each anatomical location on both sides of the animal be available for comparison. Therefore, any animals that failed placement were either partially excluded (one side excluded) or completely excluded (both sides).

### Placement Difficulty

Twenty-two data points were available for analysis at each catheter location for placement difficulty. Difficulty scores for each site were reported in Table 3. Normalcy was not achieved. There was a statistically significant difference in difficulty score between all four anatomical locations (*p* ≤ 0.0001). A *post-hoc* analysis Dunn's multiple comparison test revealed that the ileum had a significantly different difficulty score when compared to the femur and humerus (*p* ≤ 0.0001).

**Table 3:** Placement difficulty scores.

	**Humerus**	**Ileum**	**Tibia**	**Femur**
Number of values (*n*+)	22	22	22	22
Minimum	1.0	1.0	1.0	1.0
25% percentile	1.0	2.0	1.0	1.0
Median	1.0	3.0	1.5	1.0
75% percentile	1.0	5.0	3.250	1.25
Maximum	3.0	5.0	5.0	3.0

### Placement Time

The number of data points used for placement time varied due to variable success rates between anatomical locations. Placement times for each anatomical location are reported in Table 4. Normalcy was not achieved. There was a statistically significant difference between all four anatomical locations in placement time (*p* ≤ 0.05). A *post-hoc* analysis revealed that there was a significant difference in placement time between the femur and ileum (*p* ≤ 0.05).

**Table 4:** Placement time between various anatomical sites.

	**Humerus**	**Ileum**	**Tibia**	**Femur**
Number of values	18	16	18	20
Minimum (s)	12.71	13.35	13.33	13.80
25% percentile (s)	17.29	25.75	16.46	16.25
Median (s)	23.00	31.00	21.65	21.71
75% percentile (s)	31.25	61.75	40.42	25.05
Maximum (s)	55.35	127.00	95.00	117.00

### Gravity Infusion Rates

There were 14 data points used for analysis of gravity infusions. The gravity infusion rates are reported in Table 5. Normalcy was achieved at all four anatomical locations. There was a statistically significant difference in gravity infusion rates between anatomical sites (*p* ≤ 0.0004). A Tukey multiple comparison *post-hoc* analysis revealed a statistically significant difference in flow rates between the humerus and tibia (*p* ≤ 0.01) and between the tibia and femur (*p* ≤ 0.001).

**Table 5:** Gravity infusion rates.

	**Humerus**	**Ileum**	**Tibia**	**Femur**
Number of values	14	14	14	14
Mean (mL/kg/min)	0.9405	0.5295	0.2948	1.029
Standard deviation (mL/kg/min)	0.6045	0.3176	0.2927	0.6646

### Pressure Infusion Rates

There were 14 data points used for analysis of pressure infusions. The pressure infusion rates are reported in Table 6. Normality was achieved at all four anatomical locations. There was a statistically significant difference in pressure infusion rates between anatomical sites (*p* ≤ 0.0001). A Tukey multiple comparison *post-hoc* analysis revealed a statistically significant difference in flow rates between the humerus and tibia (*p* ≤ 0.0001), the femur and the tibia (*p* ≤ 0.0001), and between the ileum and the tibia (*p* ≤ 0.01).

**Table 6:** Pressure infusion rates.

	**Humerus**	**Ileum**	**Tibia**	**Femur**
Number of values	14	14	14	14
Mean (mL/kg/min)	2.068	1.515	0.6049	2.102
Standard deviation (mL/kg/min)	0.8707	0.5951	0.4942	0.7135

## Discussion

The ideal site of intraosseous access should have a high first attempt success rate, short placement time and allow rapid infusion of fluids for resuscitation. Results of the current study suggest the humerus and femur may be the preferred sites for IO catheter placement in adult dogs due to the high success rate, short placement time, and rapid infusion rates, particularly when compared to the wing of the ileum and tibia. This is in contrast to both veterinary and human literature, where the tibia is often described as the site of choice for intraosseous catheter insertion, primarily due to its easily identifiable landmarks (5).

Similar results have been demonstrated in a variety of species. A human cadaver study demonstrated superior flow rates in the humerus and sternum when compared to the tibia (13). Although identified as one of the most optimal infusion sites in human literature (13), the sternum was not utilized in this study. The presence of sternebrae in veterinary patients as compared to the single sternum in humans, may have unforeseen anatomical and physiological implications for IO catheter placement in dogs. In a porcine study, it was demonstrated that the distal femur and proximal humerus could facilitate faster flow rates than the tibia and malleolus in piglets (12). Another porcine study looking at adult pigs demonstrated faster infusion rates in the proximal humerus when compared to the tibia (14). Interestingly, flow rates in the humerus were statistically greater compared to the tibia. There was no statistically significant difference between the femur and the tibia in this study comparing adult pigs.

There are a variety of variables that can influence intraosseous fluid rate rates, which may explain some of the differences in flow rates obtained in the current study. Warren et al. demonstrated that hypovolemic patients have a statistically significantly reduction in flow rates when compared to euvolemic piglets (12), but was not found to be clinically significant. Perhaps one of the most important variables to consider when placing an intraosseous catheter is the circumference of the bone where the catheter is being placed. It was demonstrated in the human emergency department that EZ-IO catheters placed in the proximal tibia could facilitate statistically significant greater flow rates when compared to EZ-IO catheters placed in the distal tibia (with gravity and pressure infusion). Bones with a greater cross-sectional area provide less resistance to fluid flow when compared to more narrow bones. As stated by Poiseuille's Law, resistance of flow is directly proportional to radius to the fourth power and inversely proportional to the length. This theory is consistent with findings from our data. The femur and humerus had a much wider circumference when compared to the tibia. We postulate that this is due to the narrow internal radius of the tibia when compared to the femur and humerus. In addition to having slower flow rates, the tibia in smaller animals (dogs <15 kg) demonstrated a higher incidence of extravascularization into the subcutaneous space during high pressure infusion.

The ideal site of intraosseous catheter placement should have easy to identify landmarks and minimal subcutaneous tissue so that the bone surface easily palpated and the risk of injury to vital structures is minimized during catheter placement. For placement difficulty, the ileum had a significantly higher difficulty score when compared to all other anatomical locations. Additionally, the ileum had the longest average time for successful placement and the highest number of placement failures. Numerus difficulties were encountered while placing intraosseous catheters within the ileum. Dogs with even moderate body condition or muscle condition made it difficult to palpate the landmarks to orientate the operator. Additionally, the curved angle of the ileum required specific orientation of the EZ-IO gun. There were numerous occasions when the EZ-IO initially appeared to make good contact with bone, but ultimately resulted in failed placement.

Despite having easy to identify and easily accessible landmarks, there were numerous failed attempts with IO catheter placement in the proximal tibia. This may be due to the smaller cross-sectional area of the proximal tibia compared to the cross-sectional area of the distal femur and proximal humerus. Several attempts that were deemed a failure due to the IO catheter being advanced through both cortices of the tibia. Pressurized infusion in two animals resulted in fluid accumulation within the subcutaneous space despite no fluid accumulation in subcutaneous space being appreciated under gravity infusions. Both of these incidences occurred in dogs <15 kg (dog 7 and dog 10). The development of subcutaneous fluid accumulation during pressurized infusions may be the result of the IO catheter being incorrectly placed or a result of the tibia not tolerating pressurized infusions.

An additional factor to consider between different anatomical locations is time for infusions to reach the heart. Although the femur and humerus appear to be similar with regards to IO catheter placement ease, time and flow rates, the humerus may be preferred to the femur in certain situations, such as cardiac arrest. It has been demonstrated in human cadaver studies that tracer dyes injected through the IO space of the sternum reached the heart faster when compared to dye injected into the tibia (18). A recent porcine study demonstrated that dye injected into an IO catheter placed in the proximal humerus of a cardiac arrest model reached the heart as quickly as dye injected into a jugular IV catheter during active external chest compressions (19). In this study, there was no significant difference in time for contrast infusions to reach the heart between humeral IO catheters, jugular IV catheter, and cephalic IV catheter. This may be another reason an IO catheter placed into the proximal humerus is preferred over other sites in the current study. Future evaluation comparing time for infusions to reach the central circulation between other IO catheter placement sites would allow further recommendations for the optimal placement site.

The trochanteric fossa of the femur is a commonly used location for IO catheter placement in exotic and pediatric patients. However, there are important anatomical structures such as the sciatic nerve which have the potential to be damaged during placement of an IO catheter in this location. Additionally, there can be variable amounts of soft tissue covering the trochanteric fossa which can make landmark identification and successful placement of an IO catheter difficult. In was found that some dogs required a longer 45 mm IO catheter (as opposed to the 25 mm IO catheter used at all other sites) for successful placement within the trochanteric fossa. To reduce potential confounders such as different sized IO catheters being used at different anatomical sites, the trochanter fossa was not used as a location for IO catheter placement in this study. Instead, the distal lateral femur as selected as an IO catheter insertion site. This novel placement site has less soft tissue covering when compared to the trochanter fossa and the distal femoral condyles have easily palpable landmarks.

There are a number of limitations in the current study. First, the most important factor to address is the relatively small population size of animals recruited into this study, which may have resulted in type II error. The same IO catheters were used on both the right and left side of the canine cadavers. Repeated use of these catheters may have resulted in alterations in the ease of placement as well as flow rates as the study progressed. This study was performed completed by two different intraosseous operators, which may have introduced confounding and conflicting results as operators may have had different experience and preference for IO catheter placement. Additionally, as the operators became more confident with IO catheter placement, they may have developed a preference for anatomic sites in which to place IO catheters. This preference may have unintentionally led to bias between anatomical sites. Finally, as this study was performed in canine cadavers, factors such as patient handling and comfort cannot be assessed. Placement of an IO catheter as well high-pressure infusions through an IO catheter can be extremely painful. These procedures may not be tolerated by a conscious animal. The use of systemic analgesia and local anesthetics (topical and intraosseously) are often required to facilitate placement and during fluid administration. Other factors such as a fracture at the target bone, previous IO attempts at the target bone or infection at the site of insertion will also limit placement of an IO catheter (20). If IO catheter placement is not tolerated, alternative techniques for vascular access such as venous cut down, ultrasound guided placement, or jugular catheter placement may be required.

To the author's knowledge, this is the first study looking at ease of placement and fluid flow rates in various anatomical locations in canine cadavers. Future studies should address whether species (i.e., cats), age, hydration status or disease process affect IO fluid flow rates. As this study was performed on canine cadavers, further studies on live animals will need to be conducted to ensure these results are consistent.

These results demonstrate that in canine cadavers, the humerus and femur have high first attempt success rates and reduced time for placement compared to the tibia and ileum. Additionally, both the femur and humerus can facilitate fluid flow rate significantly greater than that of the tibia. The clinical implications suggest that the femur and humerus may be the preferred site for intraosseous catheter placement in times of emergency resuscitation or cardiac arrest.

## Data Availability

The datasets generated for this study are available on request to the corresponding author.

## Ethics Statement

The animal study was reviewed and approved by University of Calgary's Veterinary Science Animal Care Committee (AC-16-0216). Written informed consent was obtained from the owners for the participation of their animals in this study.

## Author Contributions

JL was responsible for majority of data collection and reporting. He wrote the research article. AB was responsible for data collection and reporting. He wrote an initial draft of the abstract. SB was responsible for creating this project as well as applying for funding. He provided substantial support during both the data collection as well as the writing of the paper. AA was responsible for creating this project as well as applying for funding to support this project. She had significant contributions to the editing and writing of this paper.

### Conflict of Interest Statement

The authors declare that the research was conducted in the absence of any commercial or financial relationships that could be construed as a potential conflict of interest.
